# Development of tryptophan metabolism patterns to predict prognosis and immunotherapeutic responses in hepatocellular carcinoma

**DOI:** 10.18632/aging.204928

**Published:** 2023-08-03

**Authors:** Guo Long, Dong Wang, Jianing Tang, Weifeng Tang

**Affiliations:** 1 Department of Liver Surgery, Xiangya Hospital, Central South University, Changsha 410008, Hunan, China; 2 National Clinical Research Center for Geriatric Disorders, Xiangya Hospital, Central South University, Changsha 410008, Hunan, China; 3 Liver Disease Center, The Affiliated Hospital of Qingdao University, Qingdao 266000, Shandong, China; 4 Department of Gastroenterology, The Second Hospital of Zhuzhou, Zhuzhou 412005, Hunan, China

**Keywords:** hepatocellular carcinoma, tryptophan metabolism, predicting prognosis, tumor microenvironment, immunotherapeutic responses

## Abstract

Tryptophan metabolism is associated with tumorigenesis and tumor immune response in various cancers. Liver is the main place where tryptophan catabolism is performed. However, the role of tryptophan metabolism in hepatocellular carcinoma (HCC) has not been well clarified. In the present study, we described the mutations of 42 tryptophan metabolism-related genes (TRPGs) in HCC cohorts. Then, HCC patients were well distributed into two subtypes based on the expression profiles of the 42 TRPGs. The clinicopathological characteristics and tumor microenvironmental landscape of the two subtypes were profiled. We also established a TRPGs scoring system and identified four hallmark TRPGs, including *ACSL3, ADH1B, ALDH2,* and *HADHA*. Univariate and multivariate Cox regression analysis revealed that the TRPG signature was an independent prognostic indicator for HCC patients. Besides, the predictive accuracy of the TRPG signature was assessed by the receiver operating characteristic curve (ROC) analysis. These results showed that the TRPG risk model had an excellent capability in predicting survival in both TCGA and GEO HCC cohorts. Moreover, we discovered that the TRPG signature was significantly related to the different immune infiltration and therapeutic drug sensitivity. The functional experiments and immunohistochemistry staining analysis also validated the results above. Our comprehensive analysis enhanced our understanding of TRPGs in HCC. A novel predictive model based on TRPGs was built, which may be considered as a beneficial tool for predicting the clinical outcomes of HCC patients.

## INTRODUCTION

Hepatocellular carcinoma (HCC) accounts for most primary liver cancers and ranks sixth in cancer incident cases worldwide [1, 2]. Hepatectomy and liver transplantation are considered as potentially curative treatments for early-stage HCC patients [3, 4]. As HCC is difficult to detect in the early stage and the growth rate is fast, more than half of HCC patients lose the surgery opportunity [5, 6]. Even though systemic therapies such as targeted and immune therapy are rapidly changing, the prognosis is far from satisfactory for patients with advanced HCC [7–10]. The 5-year survival rate for HCC is less than 20% [11]. Therefore, finding novel biomarkers and constructing an accurate model to improve prognostic stratification are crucial for HCC patients.

Tryptophan (Trp) is an essential amino acid [12]. Trp and its metabolites play a critical role in different biological processes, including maintaining cell growth and proliferation [13]. Trp is a cornerstone that mediates human physiological reactions to the environment, and its metabolites act as neurotransmitters and signaling molecules [14, 15]. The Trp metabolism process mainly occurs in the liver. Previous studies demonstrated the pathogenesis of colon cancer was associated with a faulty Trp mechanism [16]. In addition, a growing number of findings supported that Trp metabolites served a crucial role in immune regulation and inflammatory response [17]. Emerging studies also indicated that Trp metabolism was involved closely in the oncogenesis of HCC [18, 19].

Liver is proverbially regarded as a distinct immunological environment [20]. HCC is associated with the underlying immune process and influenced by the tumor microenvironment (TME) constituency [7]. TME is vital in the progress of HCC [21]. The immunological landscape of liver cancer is notably unique. A mass of immune cells and tumor cells constitute TME [22]. Therefore, immunotherapy is promising for the treatment of HCC. What’s more, aberrant activation of Trp metabolites resulted in the suppression of antitumor immunity since they modified the function of the immune cells [23, 24]. Trp metabolism was an essential regulator of tumor immune evasion [25]. However, few studies focused on crosstalk between Trp metabolism-related genes (TRPGs) and the TME of HCC. Therefore, profiling the characteristics of TME cell infiltration might provide a comprehensive view of HCC tumorigenesis and improve treatment strategy.

In this work, we analyzed the TRPGs alterations and TME of the HCC datasets from The Cancer Genome Atlas (TCGA) and Gene Expression Omnibus (GEO) database. 371 HCC patients were enrolled and classified into two groups by the levels of the TRPGs expression. Furthermore, we built a TRPGs model to predict the prognosis for HCC patients. We also profiled the immune landscape of HCC. Compared to the traditional clinicopathologic risk factors, the TRPGs signature was identified to be a potential prognostic model for HCC patients.

## RESULTS

### Alteration and expression analysis of TRPGs in HCC

**Figure 1:**
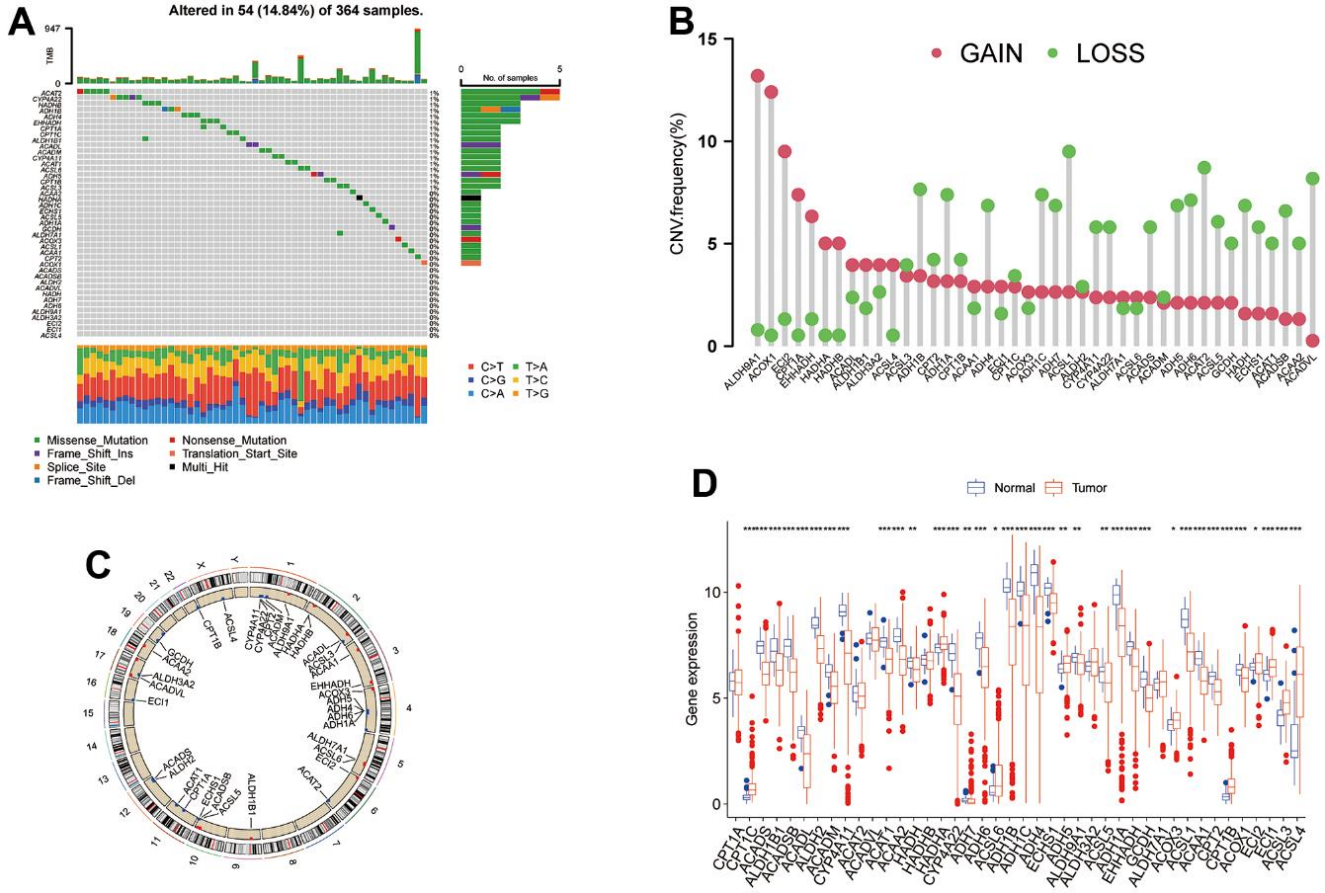
**The genetic alterations and transcriptional expression of TRPGs in HCC.** (**A**) The mutation frequencies and distribution of 42 TRPGs in the TCGA HCC cohort. (**B**) Frequencies of CNV alterations of TRPGs in HCC. (**C**) Locations of CNV alterations in TRPGs on chromosomes. (**D**) Expression distributions of 42 TRPGs between HCC tumor and normal tissues. *p<0.05, **p < 0.01, ***p < 0.001. TRPGs, tryptophan metabolism-related genes; HCC, hepatocellular carcinoma; TCGA, The Cancer Genome Atlas; CNV, copy number variation.

In this study, we identified 42 TRPGs in the HCC databases. In all 364 samples, 14.84% (54/364) of patients occurred the alteration of TRPGs. Of the 42 TRPGs, *ACAT2* and *CYP4A22* harbored the highest mutation rate, followed by *HADHB*, *ADH1B*, *ADH4*, and *EHHADH*. The missense mutation was the most frequent variant type. Besides, C > T, C > A and T> C were the most frequent Single Nucleotide Variation (SNV) types (Figure 1A). Furthermore, we analyzed the somatic copy number variations (CNVs) in 42 TRPGs (Figure 1B). *ALDH9A1*, *ACOX1*, and *ECI2* ranked the top with CNVs gain, while *ACSL1*, *ACAT2*, *ACADVL* and *ADH1B* ranked the top with CNVs loss. We also profiled the location of CNV alterations of TRPGs on chromosomes. Additionally, the expression of TRGs was investigated between the HCC tumor tissue and their corresponding normal tissues. As shown in Figure 1D, significantly differential expression was observed in these TRPGs.

### Identification of tryptophan metabolism subtypes in HCC

**Figure 2:**
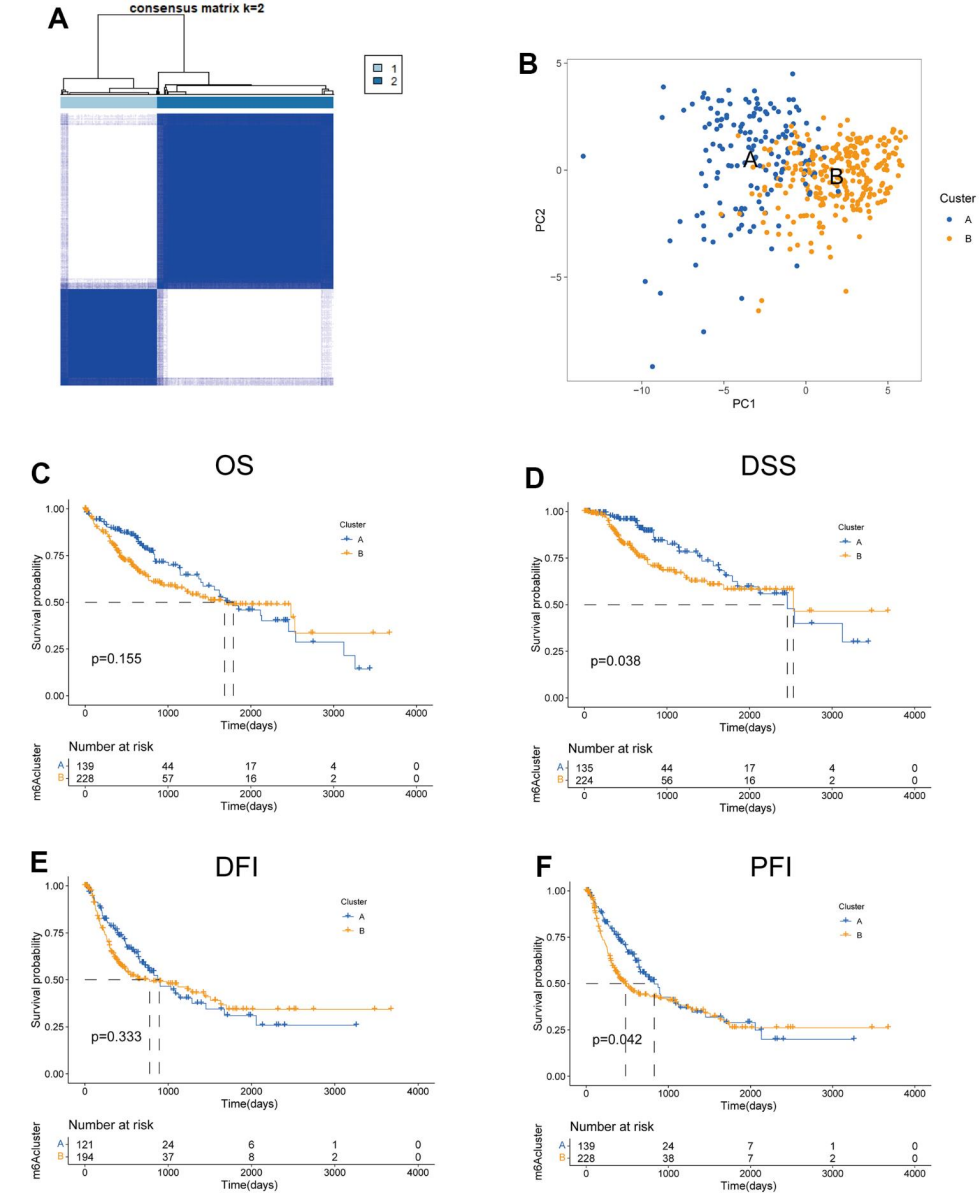
**Features of two TRPGs clusters.** (**A**) Consensus heatmap matrix and correlations areas of two clusters (k=2). (**B**) PCA analysis found the difference between the two clusters. Univariate analysis showed 42 TRPGs related to the OS (**C**), the DSS (**D**), the DFI (**E**), and the PFI (**F**). OS, overall survival; DSS, disease-specific survival; DFI, disease-free interval; DFI, progression-free interval.

To investigate the predictive value of TRPGs in HCC, we sorted HCC patients through the consensus clustering according to the expression of TRPGs. We found k = 2 was the best categorical measure to divide the entire cohort into two subtypes (Figure 2A). The principal component analysis (PCA) revealed that HCC patients were well distributed into two subtypes (Figure 2B). Subsequently, we analyzed the prognosis between the two clusters. The results showed that Cluster A had a better disease-specific survival (DSS) (*p* =0.038) and disease-free interval (DFI) (*p* =0.042) than Cluster B. There were no significant differences in the overall survival (OS) and disease-free interval (DFI) between the two subtypes (Figure 2C–2F).

**Figure 3:**
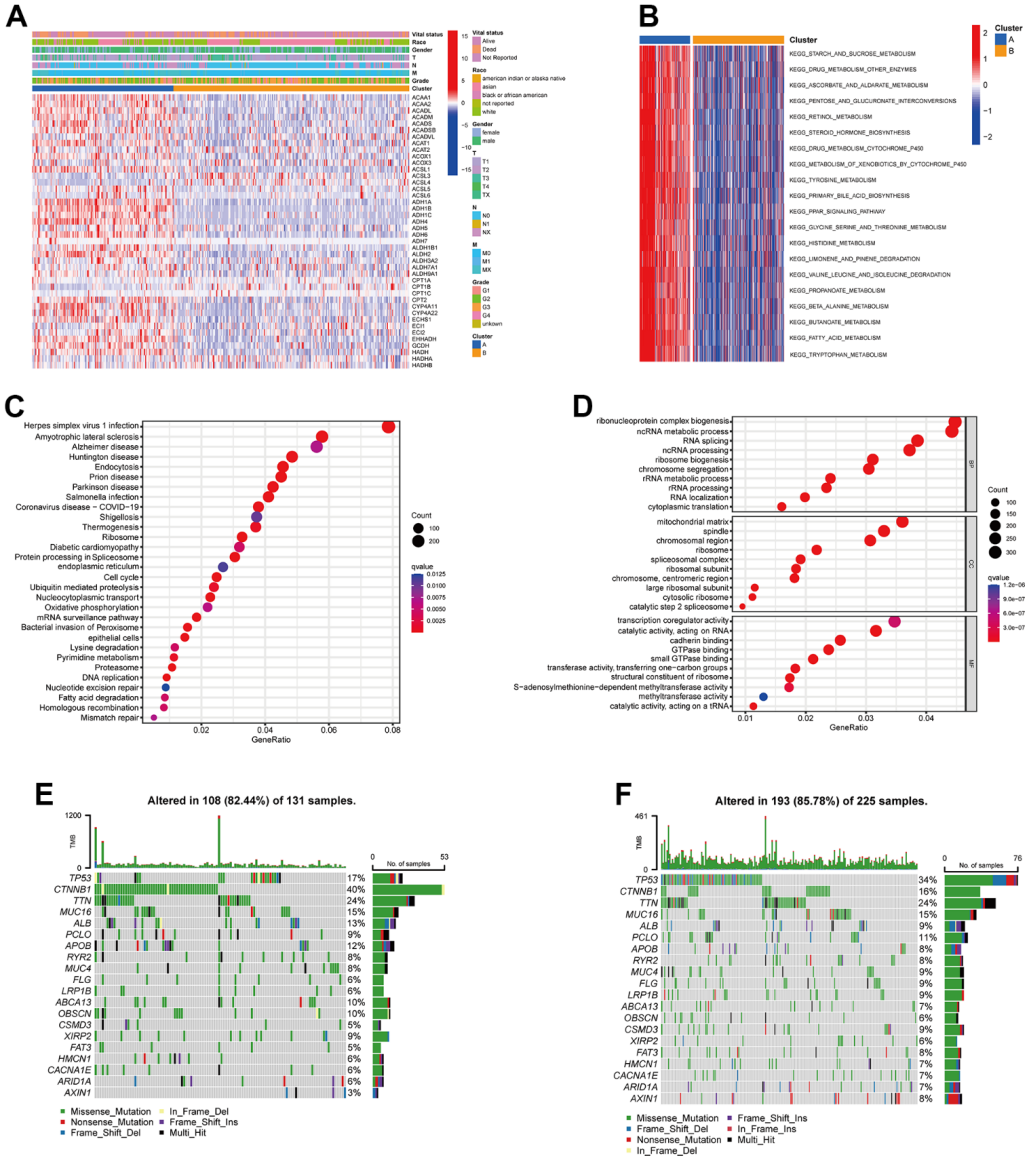
**Clinicopathological features, enrichment analysis and mutation landscape of two TRPGs clusters.** (**A**) Differences in clinicopathologic characteristics and expression levels of TRPGs between the two subtypes. (**B**) GSVA of biological pathways between two subtypes. (**C**) GO enrichment analysis showing the BP, CC, and MF of two TRGs subtypes. (**D**) The bubble plot depicted the KEGG pathway enrichment analysis of the two clusters. (**E**) Mutation landscape of TRPGs cluster A. (**F**) Mutation landscape of TRPGs cluster B. GSVA, gene set variation analysis; GO, gene ontology; BP, biological process; CC, cellular component; MF, molecular function; KEGG, Kyoto Encyclopedia of Genes and Genomes; TRPGs, tryptophan metabolism-related genes.

Next, we analyzed the clinicopathological characteristic and TRPGs expression between the two clusters. As shown in Figure 3A, most TRPGs were highly expressed in cluster A. Moreover, Figure 3B presented the results of GSVA enrichment analysis. KEGG enrichment analysis indicated TRPGs closely correlated with Herpes simplex virus 1 infection and nervous system disease (Figure 3C). The biological process (BP) showed that the TRPGs enriched their function in the ribonucleoprotein complex biogenesis, ncRNA metabolic process, and RNA splicing. The cellular component (CC) demonstrated that the TRPGs were primarily associated with the mitochondrial matrix, spindle, and chromosomal region. The TRPGs were mainly enriched in the transcription coregulator activity, catalytic activity, and acting on RNA cadherin binding for the molecular function (MF) (Figure 3D). Besides, the genetic alterations were compared between the two subtypes. The mutation frequency of two clusters were 82.44% and 85.78%, respectively. In Cluster A, *CTNNB1*(40%), *TTN* (24%) and *TP53* (17%) had the top mutation frequency. In Cluster B, *TP53*(34%), *TTN* (24%), and *CTNNB1*(16%) had the top mutation frequency (Figure 3E, 3F).

### Immune cell profile and TME of the two subtypes in HCC

**Figure 4:**
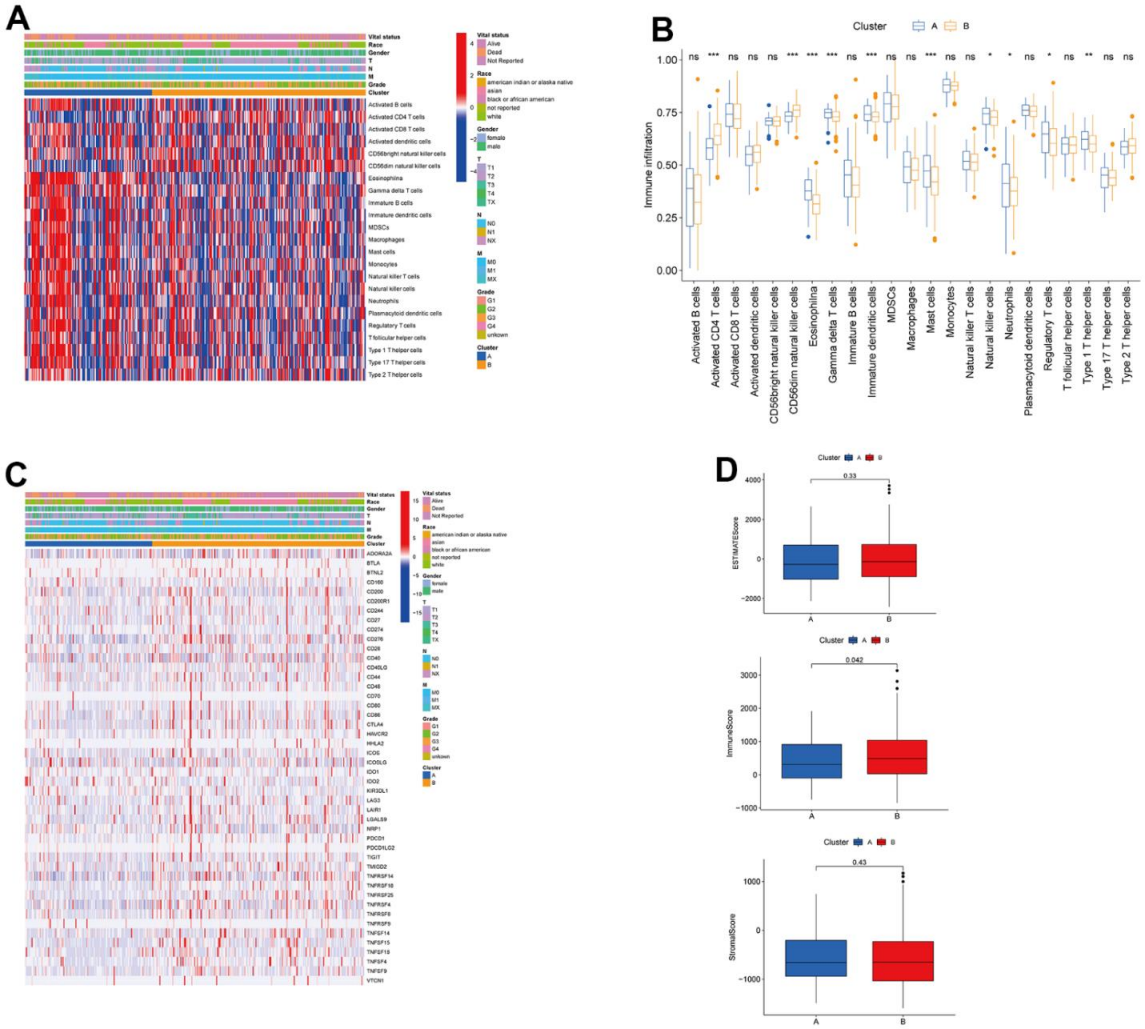
**Associations between tumor immune cell microenvironments and two HCC subtypes.** (**A**) Heatmap of the tumor-infiltrating cells and clinical features in two HCC subtypes. (**B**) Expression abundance of 23 infiltrating immune cell types in the two HCC subtypes. (**C**) Immune checkpoints heatmap between the two subtypes. (**D**) Correlations between the TME score and the two HCC subtypes. HCC, hepatocellular carcinoma; TME, tumor microenvironment.

To understand the influences of TRPGs on the TME in HCC, we explored the most common human immune cell subsets profile in the two clusters of HCC by using the ssGSEA method. The results demonstrated obvious differences of the immune microenvironment between the two groups (Figure 4A, 4B). Furthermore, the landscape of immune checkpoints was investigated between the two subgroups (Figure 4C). We found the expression of most immune checkpoints was different between the two clusters, including CD274, PDCD1 and CTLA4. Meanwhile, we also evaluated the TME score in the Figure 4D. For the TME score, the stromal or immune scores represented the content of stromal or immune cells in the TME, and the ESTIMATE scores implied aggregation of immune or stromal scores in the TME.

### Construction and validation of TRPGs risk model

**Figure 5:**
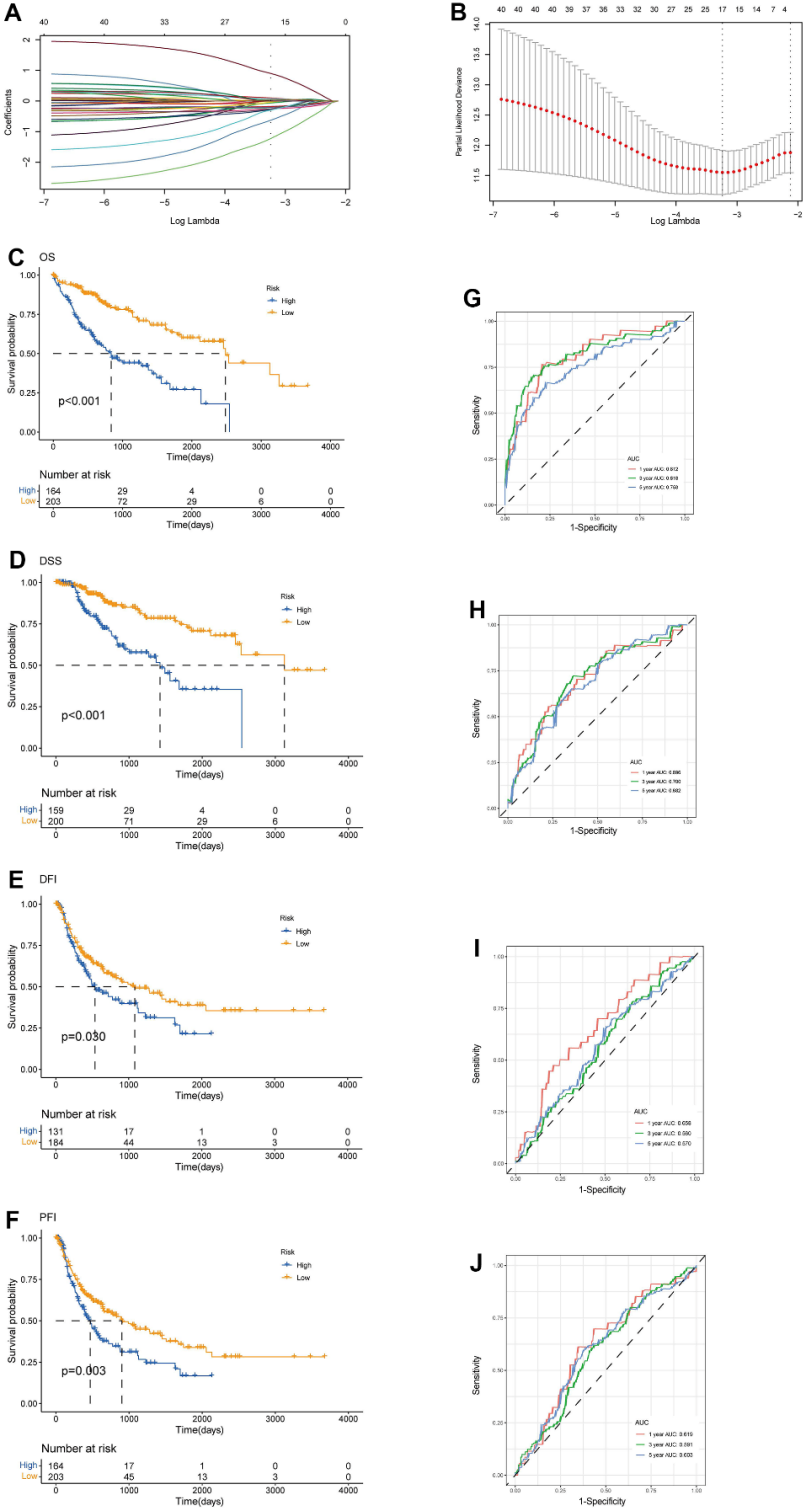
**Construction of tryptophan metabolism-related genes predictive model in the training set.** (**A**) LASSO coefficient profiles of the 42 TRPGs. A vertical line was drawn at the value chosen by 10-fold cross-validation (**B**) Ten-time cross-validation for tuning parameter selection in the lasso model. Kaplan-Meier survival analysis of the two risk subtypes according to the OS (**C**), DSS (**D**), DFI (**E**), and PFI (**F**) (log-rank tests, p < 0.01). ROC curves to predict the sensitivity and specificity of 1-, 3-, 5-year survival rates according to the risk score based on the OS (**G**), DSS (**H**), DFI (**I**), and PFI (**J**). LASSO, least absolute shrinkage and selection operator; OS, overall survival; DSS, disease-specific survival; DFI, disease-free interval; DFI, progression-free interval; ROC, receiver operating characteristic.

**Figure 6:**
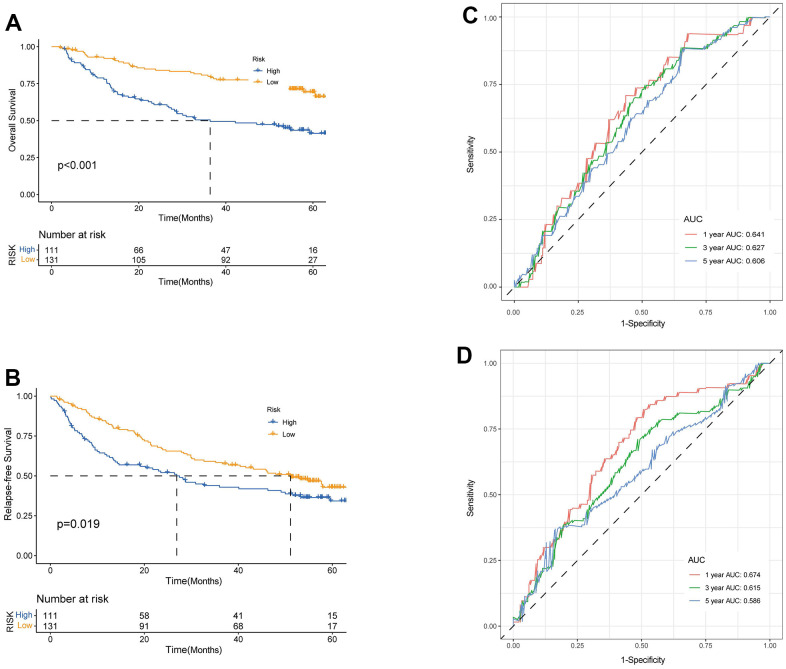
**Validation of prognostic model based on TRPGs.** Kaplan-Meier survival analysis of high- and low-risk groups in validation dataset GSE14520 (**A**), OS. (**B**), RFS (log-rank tests, p <.001). The receiver operating characteristic curve for predicting 1-year, 3-year, and 5-year OS (**C**) and RFS (**D**) of HCC patients in GSE14520. TRPGs, tryptophan metabolism-related genes; HCC, hepatocellular carcinoma.

**Figure 7:**
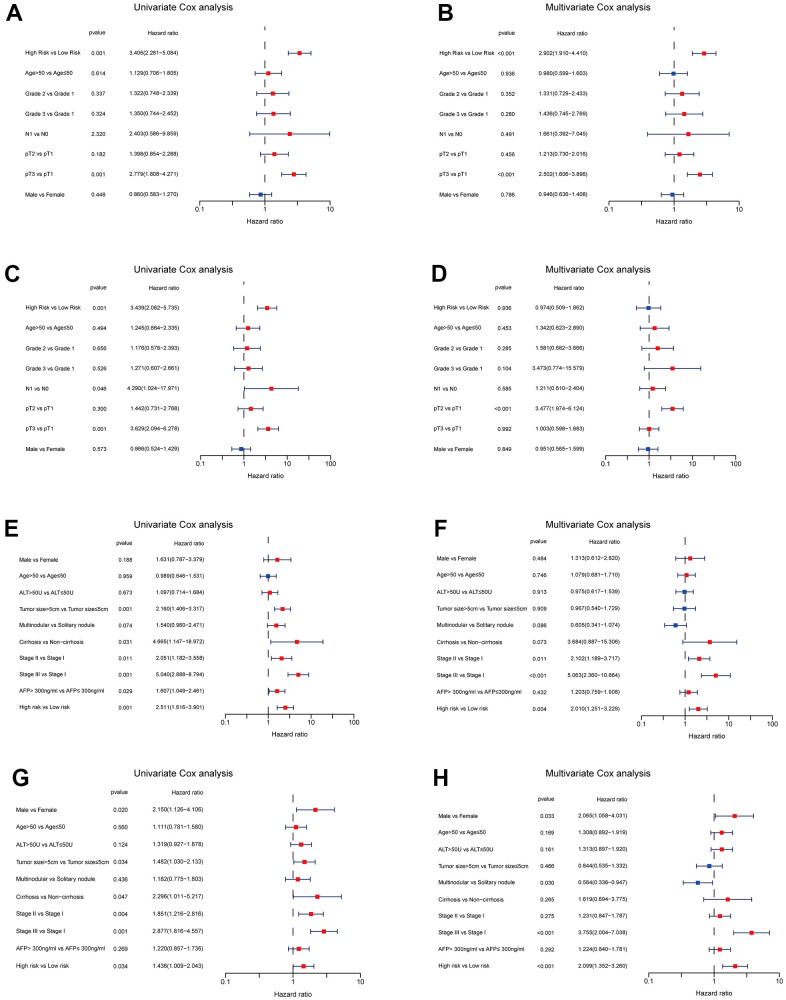
**Independent prognosis analyses of TRPGs risk model in TCGA and GES14520 HCC cohorts.** (**A**, **B**) Univariate and Multivariate Cox regression of risk score based on OS in TCGA HCC cohort. (**C**, **D**) Univariate and Multivariate Cox regression of risk score based on RFS in TCGA HCC cohort. (**E**, **F**) Univariate and Multivariate Cox regression of risk score based on OS in GSE14520 HCC cohort. (**G**, **H**) Univariate and Multivariate Cox regression of risk score based on RFS in GSE14520 HCC cohort. TRPGs, tryptophan metabolism-related genes; HCC, hepatocellular carcinoma; TCGA, The Cancer Genome Atlas; OS, overall survival; DSS, disease-specific survival; RFS, relapse-free survival.

We enrolled the all 42 TRPGs to identify prognostic genes in the training set. Based on the optimal cut-off of each gene, all HCC patient was stratified into the two groups. As presented in Supplementary Figure 1A, we identified that 24 TRPGs were closely associated with the OS. There are 2 TRPGs were related with poor OS. The others were associated with a favorable OS in HCC patients. At the same time, similar results were found in the DSS, DFI, and PFI (Supplementary Figure 1B–1D). Then, a Lasso-penalized Cox analysis with 10-fold cross-validation was performed to narrow the genes. 14 genes were identified for the prediction of the OS. Next, a stepwise multivariate Cox regression analysis was performed, and four genes were finally identified as prognostic genes to construct a predictive model (Figure 5A, 5B). We then build a four-gene signature with two high-risk genes (ACSL3 and HADHA) and two low-risk genes (ADH1B and ALDH2). The risk score of each HCC patient was calculated according to the following formula: Risk score = (0.18* expression of ACSL3) + (0.15* expression of HADHA) + (−0.2* expression of ADH1B) + (−0.22* expression of HADHA). Subsequently, HCC patients were sorted into a high-risk group and a low-risk group based on the best cut-off of the risk score. The high-risk group had a significantly poorer OS (p <.001; Figure 5C), DSS (p <.001; Figure 5D), DFI (p = .030; Figure 5E) and PFI (p = .003; Figure 5F) compared with the low-risk group. Meanwhile, we established the time-dependent receiver operating characteristic (ROC) curve analysis. The ROC area of 1-, 3-, and 5-year survival rates of OS-related prognostic subgroups were 0.812, 0.818, and 0.753, respectively (Figure 5G). And similar results for DSS, DFI, and PFI were presented in Figure 5H–5J. Furthermore, we validated the TRPGs model in the validation set (GSE14520). Kaplan–Meier’s survival analysis showed similar results. ROC curve analysis also indicated the excellent performance of the TRPGs model in predicting the prognosis (Figure 6A–6D). In addition, the univariate and multivariate Cox proportional hazards regression analyses were used to assess the importance of TRPGs signature in prognosis. We enrolled clinicopathologic factors, including age, gender, and tumor stages, in the analyses. Results (Figure 7A–7H) demonstrated that TRPGs were a significant predictive factor for OS and RFS both in univariate and multivariate analysis (p<0.05).

### Evaluation of TME and immune checkpoints in TRPGs risk models

**Figure 8:**
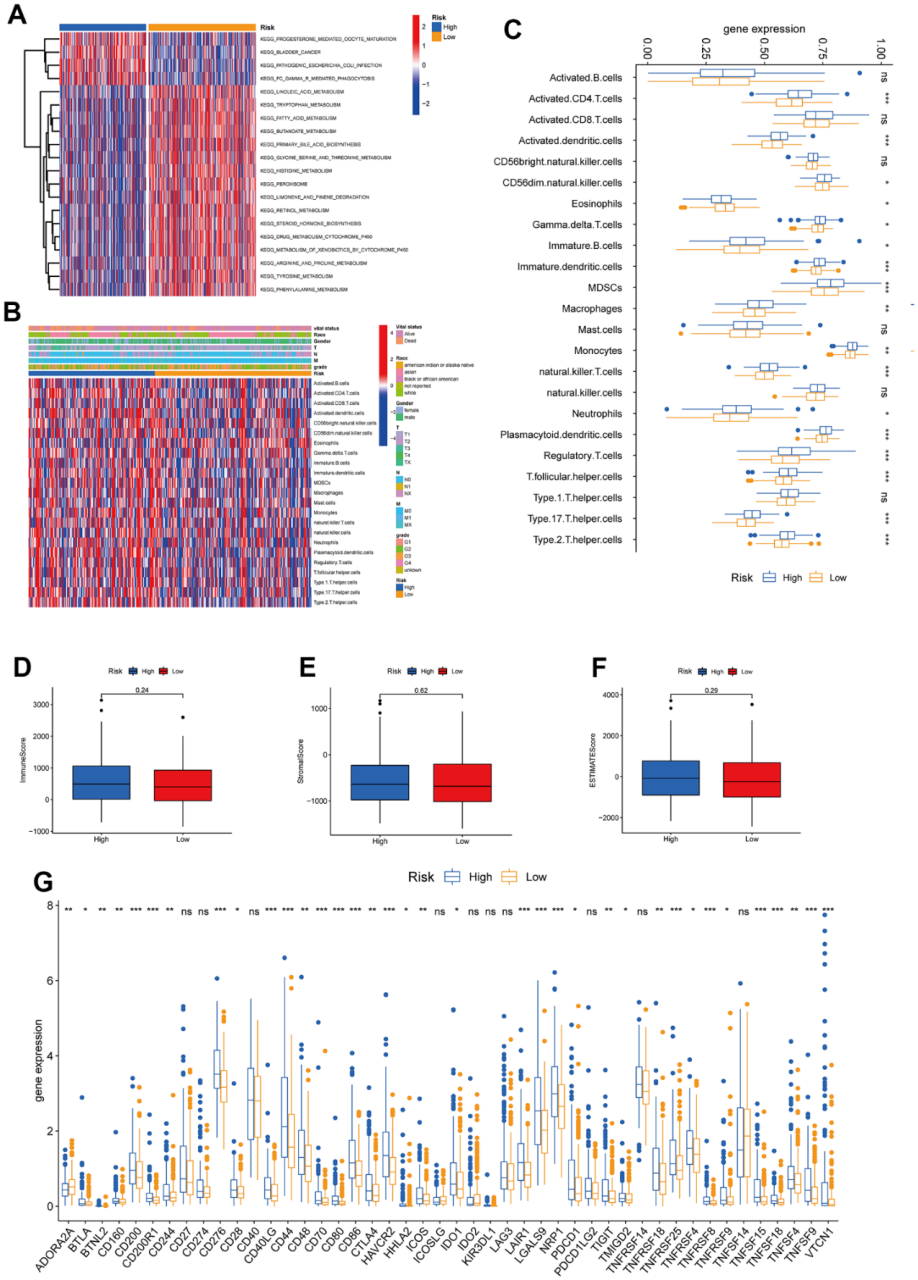
**Correlations of tumor immune cell microenvironments and two TRPGs prognostic subtypes.** (**A**) GSVA of biological pathways between two risk groups, in which red represent activated and blue inhibited pathways, respectively. (**B**) Heatmap of the clinicopathologic characteristics and tumor-infiltrating cells in the two risk groups. (**C**) Expression abundance of 23 infiltrating immune cell types in the two risk subtypes. (**D**–**F**) Correlations between the TME score and the two risk subtypes. (**G**) Expression of immune checkpoints between the two risk subtypes. *p<0.05, **p < 0.01, ***p < 0.001. TRPGs, tryptophan metabolism-related genes; GSVA, gene set variation analysis; TME, tumor microenvironment.

In this section, we analyzed the immune cell distribution and expression of checkpoints between the two TRPG risk groups. We also performed the GSVA enrichment analysis to explore the abnormal pathways. The results indicated that the functional enrichment was individual of each subtype. The high-risk group was enriched in pathways including FC Gamma R mediated phagocytosis and pathogenic Escherichia coli infection. In contrast, the low-risk group was associated with amino acid metabolism pathways (Figure 8A). Besides, we explored the relationship between the TRPGs risk model and the TME signature (Figure 8B, 8C). The infiltration level of activated CD4 T cells, activated dendritic cells, CD56dim natural killer cells, gamma delta T cells, immature B cells, immature dendritic cells, MDSCs, macrophages, monocytes, natural killer T cells, neutrophils, plasmacytoid dendritic cells, regulatory T cells, T follicular helper cells, type 17 T helper cells and type 2 T helper cells were obviously increased in the high-risk group. The infiltration level of eosinophils reduced in the high-risk group. Furthermore, the TME score was investigated in the two risk groups. However, the immune score, the stromal score, and ESTIMATE score were not significantly different between the two groups (Figure 8D–8F). Next, the profile of immune checkpoints was explored between the two subtypes. As shown in Figure 8G, PDCD1 and CTLA4 were highly expressed in the high-risk group.

### Analysis and validation of the four TRPGs used for the prognostic signature

**Figure 9:**
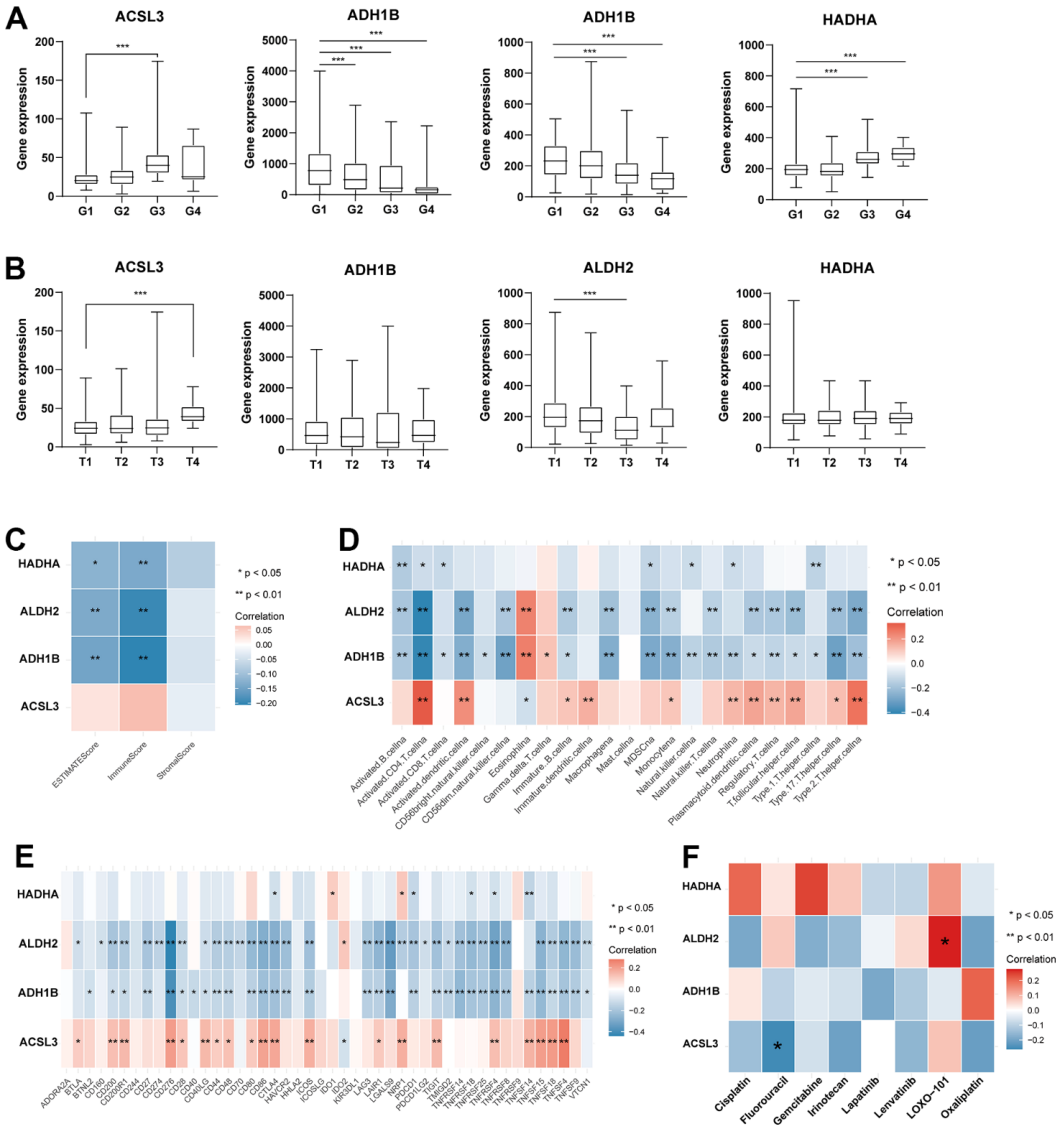
**Analysis of four TRPGs for the prognostic signature, and their correlations of tumor immune infiltrating cells and therapeutic drugs.** (**A**)The boxplot showed the relationship among *ACSL3, ADH1B, ALDH2,* and *HADHA* expression and grade stratification. (**B**) The boxplot depicts the correlation of *ACSL3, ADH1B, ALDH2,* and *HADHA* expression and T stage. (**C**) The correlation of five TRPGs and TME score. (**D**) The relationship between five TRPGs and 23 activated immune cells. (**E**) The correlation of four TRPGs and immune checkpoints. (**F**) The relationship between four TRPGs and common therapeutic drugs for HCC. *p<0.05, **p < 0.01. TRPGs, tryptophan metabolism-related genes; TME, tumor microenvironment.

We further analyzed the expression levels of four prognostic TRPGs in HCC patients. The results (Figure 9A, 9B) indicated that *ACSL3*, *ADH1B*, *ALDH2*, and *HADHA* had strongly associated with the tumor grade. Significantly, *ACSL3* and *HADHA* were upregulated in advanced tumor grade. In contrast, *ADH1B* and *ALDH2* were upregulated in the early stage of tumor grade. We then evaluated the correlation between TME score and the four TRPGs. It was presented that *ADH1B*, *ALDH2*, and *HADHA* were negatively correlated with the TME scores (Figure 9C). Besides, the relationship between the four TRPGs and immune infiltrating cells was analyzed. As shown in Figure 9D, *HADHA*, *ALDH2* and *ADH1B* were almost negatively associated with most immune cells, except for eosinophils. On the contrary, *ACSL3* was positively correlated with various immune cells. Similarly, *ADH1B*, and *ALDH2* were negatively associated with many of immune checkpoints. *ACSL3* were positively related to a great proportion of immune checkpoints, except for *IDO2* (Figure 9E). Moreover, the relationship of four prognostic *TRPGs* with the sensitivity of common therapy drugs was explored (Figure 9F). We surprisingly discovered that *ALDH2* was positively correlated to the response of LOXO-101. *ACSL3* was negatively correlated to the therapeutic effect of fluorouracil, indicating that the TRPGs may influence the therapeutic efficacy of some certain drugs in HCC.

**Figure 10:**
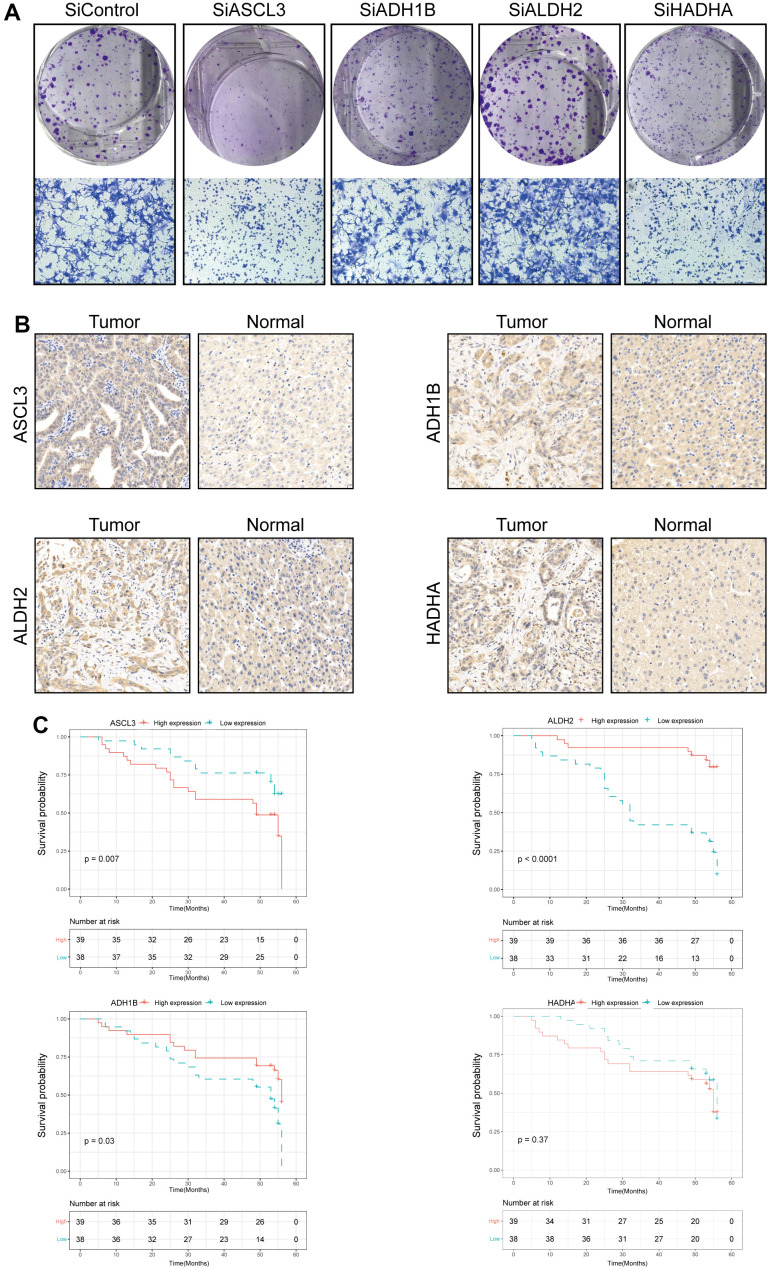
**Validation of the four prognostic genes in functional analysis and clinical data.** (**A**) The colony formation and migration analysis of LM3 cell depletion with *ACSL3, ADH1B, ALDH2,* and *HADHA*. (**B**) Immunohistochemistry analysis of *ACSL3, ADH1B, ALDH2,* and *HADHA* in HCC tissues. (**C**) The high expression level of *ACSL3* and the low expression level of *ALDH2* and *HADHA* were associated with a poor prognosis. The Cox proportion hazards model was used to understand the significance between the two groups.

In addition, we validated the biological functions of four prognostic TRPGs in HCC cell line. We used the western blotting analysis to indicate the knockdown effect of the siRNAs of ACSL3, ADH1B, ALDH2, and HADHA (Supplementary Figure 2A). The colony formation assay exhibited that depletion of *ACSL3* and *HADHA* suppressed proliferation capacity of LM3 cells, while depletion of *ALDH2* promoted the colony formation ability. The depletion of *ADH1B* didn’t influence the colony formation ability remarkably. Consistently, the similar results showed in cancer cell migration (Figure 10A). Meanwhile, we performed IHC analysis in 77 HCC patients (Supplementary Figure 2B). Results of IHC analysis demonstrated that *ACSL3*, and *HADHA* were highly expressed in HCC tissues. On the contrary, *ADH1B* and *ALDH2* were highly expressed in adjacent normal tissues (Figure 10B). Survival analysis indicated that high *ACSL3* protein levels and low *ADH1B* or *ALDH2* protein levels were associated with poor prognosis (Figure 10C).

## DISCUSSION

HCC was a highly aggressive tumor which is induced by muti-factors [26]. It occurred in patients with underlying liver disease, mainly caused by hepatitis virus infection or alcohol abuse [27]. However, the prevalence of nonalcoholic fatty liver disease (NAFLD), which in conjunction with metabolic syndrome and obesity significantly increases the risk of NAFLD, was poised to become a leading cause of hepatocellular carcinoma worldwide [28, 29]. Trp metabolism played a vital role in substance catabolism. Recently, many research have reported on the diverse functions of Trp metabolites in neurophysiology and immunology [12, 30]. Moreover, a growing number of studies indicated disturbance of Trp metabolites involved deeply in disease development and tumorigenesis [31]. Therefore, profiling the molecular features of TRPGs and their influences on the tumor immune environment was crucial in HCC.

In this study, a landscape of TRPGs and TME characteristics in HCC by using high-throughput expression profilin was constructed. We had further classified the samples into two distinct molecular subtypes based on the expression levels of 42 TRPGs. The two molecular subtypes showed different immune characteristics of TME. Patients of Cluster A had a better prognosis. Besides, we established an effective prognostic TRPGs risk model that included four TRPGs (*ACSL3, ADH1B, ALDH2, and HADHA*). Our results revealed that the risk model achieved a good discrimination performance. Meanwhile, the multivariate Cox analysis indicated this TRPG-related signature was an independent risk factor along with tumor size and tumor stage for HCC prognosis. Moreover, we analyzed the association between four TRPGs and TME. The results showed a close correlation between the four TRPGs and immune cell infiltration as well as immune checkpoints in HCC. Our TRPG prognostic model may offer new insights for targeted and immune therapy.

Currently, the prognosis of HCC is still far from satisfactory. Thereby, constructing an accurate model to identify the HCC patients with high risk was necessary. Metabolic syndrome was closely involved in the occurrence of HCC [32]. In the present study, HCC patients were clearly stratified into low- or high-risk clusters via the expression of TRPGs. This model facilitated the identification of high-risk groups for hepatocellular carcinoma (HCC) and enabled prompt implementation of effective personalized interventions by clinicians. Meanwhile, our model demonstrated high accuracy and sensitivity in both the training and validation cohorts, indicating its robust representativeness and stability. Our TRPG-related signature provided a brand-new perspective for predicting the HCC prognosis, especially in the traditional biomarkers such as APF or des-gamma-carboxy prothrombin (DCP) were negative [33].

In addition, immunotherapy and targeted therapy had changed conventional HCC treatment [34]. However, only 15-20% of HCC patients had benefited significantly from single-agent immune checkpoint inhibitors, and biomarkers had yet to identify this group [35, 36]. The current study also revealed the landscape of TME and immune checkpoints in TRPGs risk models. Hence, our findings might identify the patients who have response to the immune therapies. What’s more, *ALDH2* had been found to have a positive correlation with the response of LOXO-101. Furthermore, there was a negative correlation between ACSL3 and the therapeutic efficacy of fluorouracil. LOXO-101 also known as Larotrectinib [37]. Recent studies demonstrated that Larotrectinib had impressive therapeutic effects to some tumor [38, 39]. Fluorouracil is a common chemotherapy drug. The latest Phase III Trial found that the combination of Oxaliplatin Plus Fluorouracil was widely used in interventional hepatic arterial infusion chemotherapy (HAIC). The treatment showed better survival results than sorafenib for advanced HCC, even with a high tumor burden [40]. The two-prognostic signature TRPGs were a good guidance for patients who need receive HAIC with Fluorouracil or targeted drug therapy. And it had good potential to optimize the cost-effective of drugs.

Amounts of research revealed that TME played a vital role in the treatment and prognosis of tumors [41, 42]. In this study, we discovered that the two TRPGs subtypes had different TME features. The immune score of subtype B exhibited a statistically significant increase compared to that of subtype A. The prognosis of the two subtypes was significantly different between two clusters. Above findings proved that TME was crucial in the immunotherapy of HCC. Trp and its metabolites were an essential part of diverse physiological processes [43, 44]. The enzymatic conversion closely associated with IDO1, IDO2 and TDO was the rate-limiting step in the tryptophan metabolism process [12]. In tumor, aberrant activation of IDO1 and TDO leads to the inhibition of anti-tumor immunity. In autoimmunity, IDO1 and TDO impaired T cells and antigen-presenting cells [24, 25, 45, 46].

In our study, *ACSL3*, *ALDH2* and *HADHA* were identified as high-risk genes for HCC. Long-chain fatty acyl CoA synthetases (*ACSLs*) facilitates intracellular metabolism by activating fatty acids [47]. Recent studies indicated that *ACSL3* was increased in tumor tissue compared with normal liver [48]. Meanwhile, the molecular target of peroxisome proliferator-activated receptor delta in HepG2 hepatoma cells was considered to be ACSL3 [49]. Aldehyde Dehydrogenase 2 Family Member (*ALDH2*) is an important member of the aldehyde dehydrogenase family [50]. Some findings stated that *ALDH2* mutations were critical in the activation of hepatocellular carcinoma carcinogenic pathways and related to immune characteristics in HCC [51]. Hydroxyacyl-CoA dehydrogenase alpha subunit (*HADHA*) is a crucial lipid metabolic enzyme which plays an important role in carcinogenesis [52]. Yang et al. stated that *HADHA* mediated lipid reprogramming to promote HCC [53]. The *ADH1B* (Alcohol Dehydrogenase 1B (class I) has been explored in many studies, which is closely associated with alcohol metabolism, liver function and cancer [54]. A large cohort analysis stated that the alteration of *ADH1B* increases the risk of hepatocellular carcinoma [55]. Our findings were in line with the previous study, which suggests that these factors play a significant role in HCC development. Our risk model demonstrated a robust capability in predicting HCC prognosis and evaluating immunogenicity.

However, it was important to note that this study still had several limitations. Firstly, this study was a retrospective study. All the subjects included were from a public database, so there was inevitable selection bias. Secondly, we only constructed the TRPGs risk model and lacked real-world large sample data for verification. Finally, some crucial clinical data to validate the key prognostic features of this model, such as the evaluation of the response to immunotherapy and chemotherapy, was lacked. Therefore, a large-scale, meticulously designed and prospective study was imperative to validate our findings.

In summary, our study profiled the molecular signature of TRPGs and identified four TRPGs to construct a robust predicting model in HCC. The four prognostic genes (*ACSL3, ADH1B, ALDH2,* and *HADHA*) were strongly correlated with immune cell infiltration and prognosis of HCC patients. These findings had improved our comprehension of the tumor immune microenvironment and presented a novel advantageous tool for prognosticating the clinical outcomes of HCC patients.

## MATERIALS AND METHODS

### HCC data acquisition and processing

Gene expression data, somatic mutation data, copy number variation, and the matching clinical information of HCC were retrieved from the TCGA database and GEO database. Gene expression of 424 samples (50 normal and 374 tumor samples) from 371 patients and RNA sequencing (RNA-seq) data were derived from TCGA. Gene expression data from different samples were combined into genomicMatrix; all data was then log2 transformed. The RNA expression data was normalized using an average standard deviation of 1. The clinicopathological information of the 371 patients with HCC was described in Supplementary Table 1.

### Consensus clustering analysis of TRPGs

All TRPGs were retrieved from the MSigDB (KEGG- TRYPTOPHAN-METABOLISM). After unsupervised consensus clustering of TRPGs, the patients were divided into distinct molecular subtypes by using the R package “ConsensusClusterPlus”. To perform the clustering, the following criteria were observed: First, the cumulative distribution function (CDF) curve exhibited a gradual and smooth increase initially. Furthermore, none of the groups had insufficient sample sizes. Lastly, the intra-group correlation was found to increase, while the inter-group correlation decreased following clustering.

### Functional enrichment analysis and construction of a TRPGs-based prognostic model

To compare biological processes between the two TRPG clusters, we conducted Gene Ontology (GO) enrichment and Kyoto Encyclopedia of Genes and Genomes (KEGG) pathway analysis using the “clusterprofler” R package. Next, we used the “surv cutpoint” function from the “survminer” R package to identify critical TRPGs in HCC specimens through optimal survival cut-off analysis. The detailed procedures of the functional enrichment analysis and model construction were described in our previous study [56].

### Characterization of the immune signature of HCC

We used the ESTIMATE algorithm to calculate immune cell abundance in high-risk and low-risk groups based on TCGA data. Additionally, we employed the ssGSEA algorithm [57] to profile the extent of immune cell infiltration within the HCC TME.

Moreover, we calculated the TME score for both subtypes using ESTIMATE (Estimation of Stromal and Immune cells in Malignant Tumor tissues using Expression Data).

### RNA inference, colony formation and migration analysis

Small interfering RNA was used to knock down the expression of *ACSL3*, *ADH1B*, *ALDH2* and *HADHA*. The details of SiRNAs are in Supplementary Table 2. Besides, SiRNA transfection was performed using Lipofectamine 2000 (Invitrogen, Carlsbad, CA, USA) following the manufacturer’s instructions. The LM3 cells were used in colony formation and migration assays. The assays were performed as previously described [56].

### Immunohistochemistry staining for HCC samples

We collected HCC tumor tissue from 77 patients at the Xiangya Hospital. All patients provided written informed consent to participate in this study. The study was approved by the Ethics Committee of Xiangya Hospital affiliated to Central South University (No. 201703377). Immunohistochemistry staining was performed following standard procedures. The primary antibodies were described in Supplementary Table 2. The expression of ACSL3, ADH1B, ALDH2, and HADHA was blindly quantified by two pathologists using histochemical score (H-score).

### Statistical analysis

We performed a Chi-Square test to analyze the differences between the two groups. By using a two-tailed log-rank test and the Kaplan-Meier curve analysis, the prognostic significance was assessed for OS, DSS, DFI, and PFI. Multivariate Cox regression analysis was performed to assess the contribution of the score associated with Trp metabolism to the predictive model of HCC by using the R package “survival”. We used the ‘survivalROC’ R package to assess the predictive accuracy of the TRPGs-related model through ROC analysis. A significance level of P < 0.05 was adopted and all tests were two-tailed. All statistical analyses were performed using R software (version 4.1.2).

## Supplementary Material

Supplementary Figures

Supplementary Tables
