# Supplementary material for: Development of tryptophan metabolism patterns to predict prognosis and immunotherapeutic responses in hepatocellular carcinoma
Source: Aging (Albany NY). 2023 Aug 3;15(15):7593–615. doi: 10.18632/aging.204928 (PMC10457071; doi:10.18632/aging.204928)
Supplement: Supplementary Figures [file aging-15-204928-s001.pdf]

SUPPLEMENTARY FIGURES

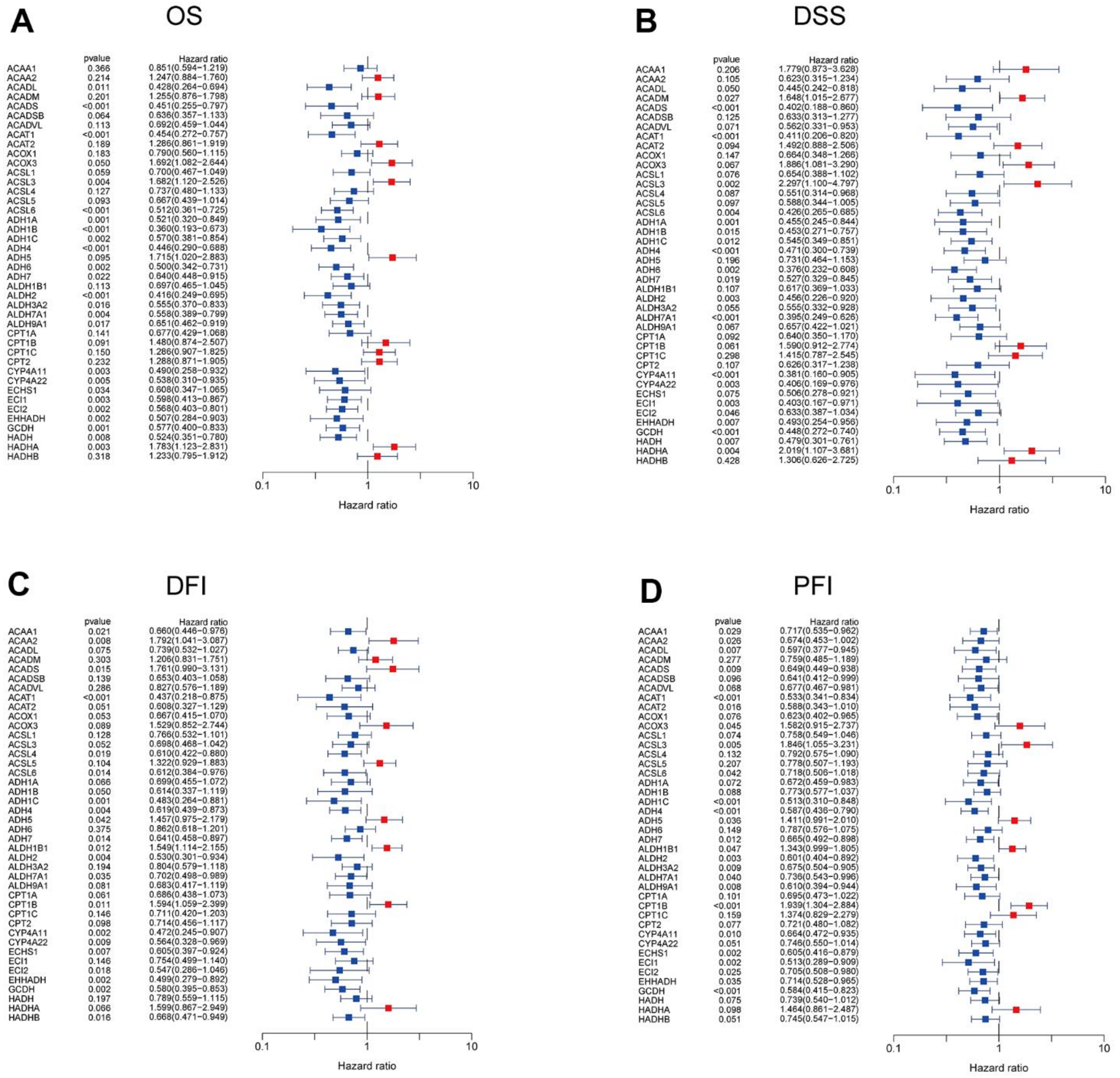

Supplementary Figure 1. The overall predictive value of 42 TRPGs in HCC patients. Univariate Cox regression analysis of 42 TRPGs associated with OS (**A**), DSS (**B**), DFI (**C**), and PFI (**D**) in HCC patients. TRPGs, tryptophan metabolism-related genes; HCC, hepatocellular carcinoma; OS, overall survival; DSS, disease-specific survival; DFI, disease-free interval; DFI, progression-free interval.

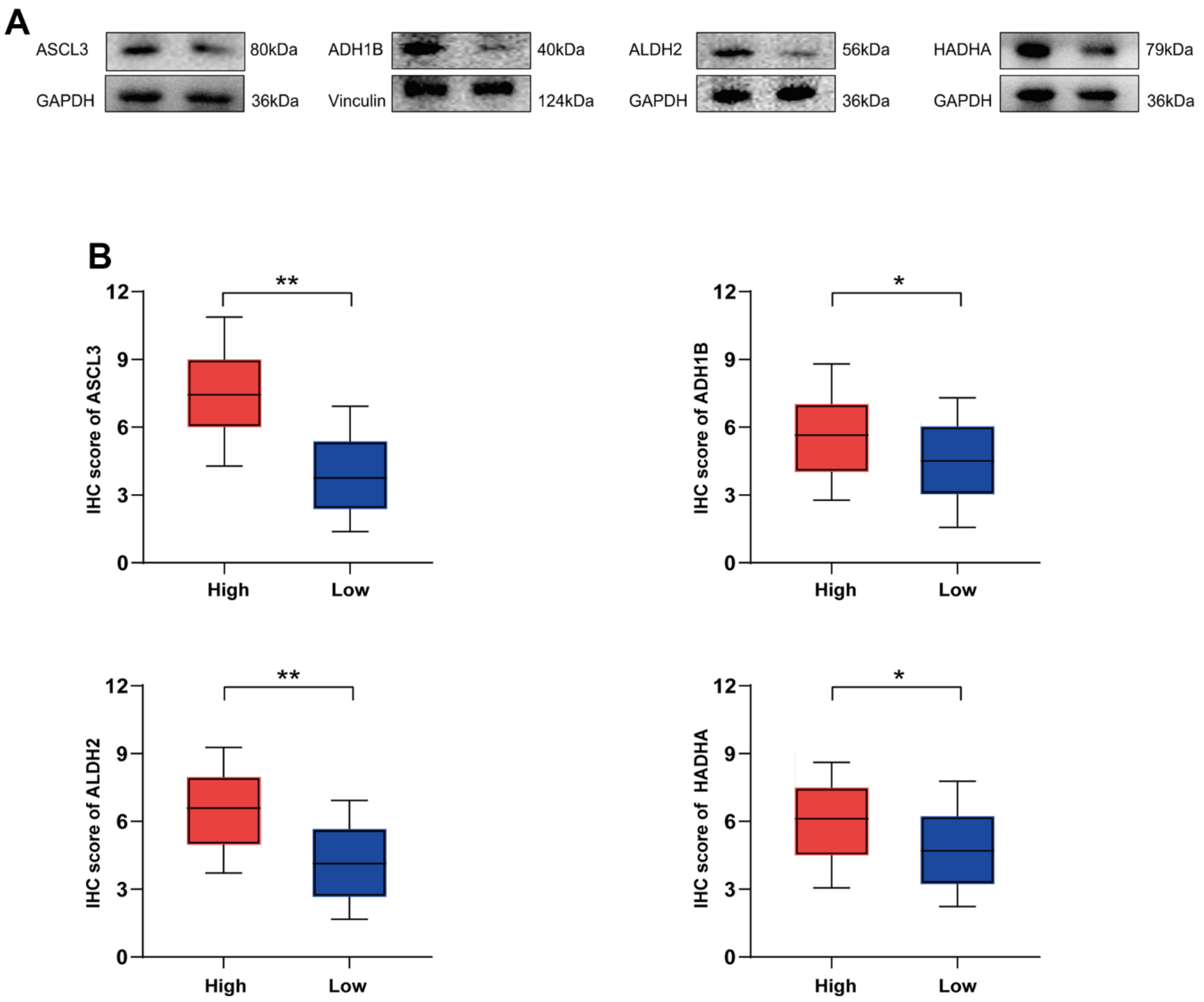

Supplementary Figure 2. (**A**) Knockdown efficiency of ASCL3, ADH1B, ALDH2 and HADHA in HCC cells. (**B**) IHC score of ASCL3, ADH1B, ALDH2 and HADHA in tumor and normal tissues.
