# Supplementary material for: Development of tryptophan metabolism patterns to predict prognosis and immunotherapeutic responses in hepatocellular carcinoma
Source: Aging (Albany NY). 2023 Aug 3;15(15):7593–615. doi: 10.18632/aging.204928 (PMC10457071; doi:10.18632/aging.204928)
Supplement: Supplementary Tables [file aging-15-204928-s002.pdf]

SUPPLEMENTARY TABLES

Supplementary Table 1. Clinicopathologic characteristics of HCC patients (n=371).

**Age at diagnosis (years)**	
≤ 50	81
> 50	290
**Gender**	
Male	121
Female	250
**T**	
T1	181
T2	94
T3	80
T4	13
TX	3
**N**	
N0	252
N1	4
NX	115
**M**	
M0	266
M1	4
MX	101
**Grade**	
G1	55
G2	177
G3	122
G4	12
unknown	5
**Vital status**	
Alive	240
Dead	130
**Median follow-up (Months)**	19.4

HCC, hepatocellular carcinoma.

Supplementary Table 2. siRNAs and antibodies used in the study.

**SiRNAs**		
ACSL3	Ruibo Biotechnology Co., Ltd. (Guangzhou, China)	siG000002181A-1-5
ADH1B	Ruibo Biotechnology Co., Ltd. (Guangzhou, China)	siG1452893545-1-5
ALDH2	Ruibo Biotechnology Co., Ltd. (Guangzhou, China)	siG000000217A-1-5
HADHA	Ruibo Biotechnology Co., Ltd. (Guangzhou, China)	siG000003030A-1-5
**Antibodies**		
ACSL3	Proteintech, Wuhan, China	20710-1-AP
ADH1B	Proteintech, Wuhan, China	17165-1-AP
ALDH2	Proteintech, Wuhan, China	15310-1-AP
HADHA	Proteintech, Wuhan, China	10758-1-AP

HCC, hepatocellular carcinoma.
